# The Rhythm Reproduction Task for children: A psychometric examination

**DOI:** 10.3758/s13428-026-03086-x

**Published:** 2026-07-07

**Authors:** Katharina Schaaf, Klaus Frieler, Franziska Degé

**Affiliations:** Max Planck Institute for Empirical Aesthetics, Grüneburgweg 14, 60322 Frankfurt am Main, Germany

**Keywords:** Rhythmic skills, Children, Pattern production, Production mode, Measurement, Reliability

## Abstract

Rhythmic ability includes various subskills, such as rhythm production and perception of beat, meter, and pattern. Among these, reproducing rhythmic patterns is a key measure of productive rhythmic skills. This study examined the psychometric properties of the Rhythm Reproduction Task for children (Jungbluth & Hafen, 2005) and an extended version including permuted item variants. A total of 151 children aged 5 to 8 completed the task across five response modalities (drumming after the experimenter, clapping, tapping, drumming after a loudspeaker, and vocal reproduction). Analyses showed appropriate item difficulty and variance, good internal consistency (*α* = .86, *ω* = .77), and strong correlations between original and permuted items (*r* = .84–.89). Structural equation modeling indicated partial metric invariance across item versions and response modalities. Results support the Rhythm Reproduction Task as a reliable measure of rhythmic pattern reproduction in children, with permuted items expanding its applicability.

## Introduction

Rhythm, with all its aspects, plays a central role in research on musical abilities. Numerous findings underscore its substantial relevance not only for music but also for cognitive development (e.g., executive functions) and social interaction (Cirelli et al., 2024; Frischen et al., 2022; Nitin et al., 2023). Rhythmic ability can be divided into several subskills (e.g., beat-related, pattern-related, meter-related), which undergo significant developmental changes throughout childhood. To investigate these developments appropriately from an early age onward, it is essential to employ high-quality measurement tools that assess the various subcomponents and apply rhythm tasks suitable for the respective age group. A promising instrument for measuring the subskill pattern production was proposed by Jungbluth and Hafen (2005), but it has not yet been validated.

The Rhythm Reproduction Task is a screening tool designed to assess rhythmic production abilities in kindergarten and primary school children. The test material consists of ten rhythm patterns that children first listen to and then reproduce. This paradigm, along with the associated rhythmic stimuli, has already been successfully employed in several studies involving children (Degé et al., 2015; Kertész & Honbolygó, 2023; Sallat, 2012; Steinbrink et al., 2019). Will (2024) even used the Rhythm Reproduction Task in different production modes (e.g., drumming, clapping, tapping), generating a dataset that allows for comparative evaluation. As part of the study, three additional permuted variants were generated for each item in order to expand the item pool and enable the creation of distinct item sets for the tested conditions. However, these alternative item versions have not yet undergone formal evaluation, and their statistical validity and comparability remain unverified. Therefore, the present study aims to systematically evaluate both the original Rhythm Reproduction Task and the expanded item pool for use under different experimental conditions.

## Rhythmic abilities

Rhythmic ability is an umbrella term encompassing multiple subskills such as beat and meter perception and production, as well as rhythmic pattern recognition and production. In general, rhythm can be understood as the temporal structure of events with well-defined onsets and separated by inter-onset intervals (IOIs). In musical contexts, the onset structure of events often exhibits certain statistical regularities, which give rise to the perception of an underlying pulse, a sensation of equal temporal spacing, commonly referred to as the beat. The beat can further be grouped into sections, each typically marked by a distinct emphasis at its beginning. A grouping of beats along with its implicit accentuation is referred to as meter. A rhythmic pattern is simply a sequence of beat-driven sound events, which may differ in duration, accentuation, and IOIs.

Rhythmic abilities, encompassing the subcomponents of beat, meter, and pattern, can be broadly categorized into perceptual and production-based abilities (Fiveash et al., 2022; Tierney & Kraus, 2015). Furthermore, empirical differences have been observed at the behavioral level between subcomponents of rhythm, such as beat-based and pattern-based rhythmic abilities (Bonacina et al., 2019). This distinction likely reflects the differing cognitive demands associated with each component. Beat-based abilities, such as synchronizing one’s movement to a steady beat, primarily rely on the anticipation of regular temporal events, a process known to be influenced by attention (Khalil et al., 2013; Puyjarinet et al., 2017). In contrast, pattern-based rhythm tasks, such as pattern reproduction, require precise timing across more complex temporal structures, along with coordinated motor control and inhibitory regulation, placing additional demands on working memory (Kim et al., 2022; Löser et al., 2024; Saito, 2001; Tierney & Kraus, 2015). For instance, studies have shown that pattern reproduction in adults is influenced by beat perception, musical training, and short-term memory, as well as by interactions among these factors, with moderate effect sizes (Grahn & Schuit, 2012). Thus, rhythmic pattern performance reflects the integrated influence of multiple cognitive and motor systems.

## Development of rhythmic abilities

The development of rhythmic abilities begins prenatally (Pino et al., 2016; Ullal-Gupta, 2013) and undergoes significant changes throughout childhood (Nave-Blodgett, 2021; Reifinger, 2006). By weeks 27–30 of gestation, the preterm infant brain exhibits neural synchronization to an induced beat, responds to deviations from isochrony, and by week 33 can detect nested rhythmic structures (meter), indicating that the neural substrate for rhythm processing is already established in utero (Edalati et al., 2023; Saadatmehr et al., 2024). Behavioral performance, in contrast, develops later during childhood. Different rhythmic abilities develop at varying rates, with perceptual abilities typically emerging earlier than productive ones. This is likely due to the developmental advantage of the auditory system over the motor system (Derri et al., 2001; Rainbow, 1981). By five months of age, infants can already distinguish simple rhythmic patterns with a salient beat structure when they hear them (Trehub & Hannon, 2008). Over the following years, the ability to discriminate rhythms continues to improve, gradually refining into adulthood (Will, 2024). In contrast, rhythm production emerges later. Around 2.5 years of age, children begin to produce regular temporal intervals during spontaneous drumming and can adjust their tempo to match an external stimulus, an early form of beat-based production (Provasi & Bobin-Bègue, 2003). However, the ability to adapt tempo develops gradually with age and remains less precise than in adults even at 12 years (McAuley et al., 2006). Pattern-based production abilities develop even later. A typical task would be reproducing simple rhythmic patterns. Performing this task is highly challenging for 4-year-olds, shows notable improvement by age 5, and continues to develop into adulthood (Reifinger, 2006).

Initial findings indicate that rhythm perception and production follow distinct developmental trajectories (Will, 2024) and may be differentially related to other cognitive and behavioral domains such as working memory, auditory attention, or motor skills (Bonacina et al., 2019). Moreover, research suggests that children’s rhythmic production is influenced by the specific motor demands of the task, with variations observed across different movement modalities, such as clapping versus tapping (Rainbow, 1981; Will, 2024). While a reliable association between rhythmic perception and production has been observed in healthy adults (Kim et al., 2024; Repp et al., 2010), this relationship remains less well studied in children. Perception-based tasks continue to dominate the assessment of rhythmic abilities in developmental research, resulting in a limited understanding of the development of rhythm production skills and their potential interaction with perceptual processes. Studies that have examined both domains in children report mixed findings (Nave-Blodgett et al., 2021; Will, 2024), which may depend on the specific tasks and modalities assessed.

## Measuring rhythmic abilities

To properly assess and compare rhythmic abilities, reliable measurement tools for the subcomponents are essential. In the past, however, these components have received varying levels of attention. The history of musicality assessment, including rhythm testing, has long been shaped by an emphasis on pattern-based auditory discrimination methods. This approach was exemplified by the very first standardized test of musicality, in which participants were required to compare rhythm patterns and judge their similarity (Seashore, 1919). Despite its highly reductionist nature, this psychophysical approach exerted significant influence on subsequent test development due to its ease of administration and scoring. Building on this foundation, perception-based musicality assessments have continued to evolve, leading to the development of tests for various age groups, such as the Primary Measure of Music Audiation (PMMA; Gordon, 1979), the Musical Ear Test (MET; Wallentin et al., 2010), and the Profile of Music Perception Skills (PROMS; Law & Zentner, 2012). Furthermore, recent approaches move beyond isolated rhythm sequences to examine rhythm perception in more naturalistic musical contexts, bringing the assessment closer to everyday listening experiences. One such example is the Beat Alignment Task, in which participants evaluate how well a beat fits with a musical excerpt (Iversen & Patel, 2008; Harrison & Müllensiefen, 2018).

Despite significant advancements in perception-based assessments, musicality remains an inherently multimodal affair that cannot be fully captured by perceptual mechanisms alone (Buren et al., 2021; Müllensiefen et al., 2014; Hallam & Prince, 2003). Consequently, in the second half of the 20th century, researchers increasingly incorporated rhythm production into their measurement approaches. As with standardized perception tests, the diagnostic goal was to develop test items that clearly define the task and, at an appropriate level of difficulty, enable verification of correct performance based on objectively assessable responses. The predominant paradigm for pattern-based production has been the reproduction of pre-recorded rhythmic patterns (Roberts & Davies, 1975; Atterbury, 1983; Drake, 1993; Smith et al., 1994), and for beat-based production, the active synchronization to an external beat (Igaga & Versey, 1978; Thackray, 1969), two tasks that are still used today (e.g., Carrillo et al., 2024; Kirschner & Tomasello, 2008; Smolej-Fritz & Peklaj, 2019). Compared to perception tests, however, there have been new challenges in performance recording. In the early stages of rhythm production assessment, the standard evaluation procedure for pattern reproduction involved assigning a score of 0, 1, or 2 to each response, depending on whether it was completely incorrect, partially correct, or fully correct. Synchronization was assessed via number of correct strokes as judged by the experimenter (Igaga & Versey, 1978). This type of evaluation has been widely criticized, as auditory-based assessments conducted solely by experimenters may lack sufficient precision and objectivity when measured against established diagnostic standards (Repp et al., 2005). In recent decades, substantial advancements have been made in this regard. Rhythm performance is now increasingly captured not only through observational methods but also via Musical Instrument Digital Interface (MIDI) (Repp et al., 2005) or sophisticated recording systems (Will, 2024). These developments have introduced new challenges for data processing, such as the application of circular statistics for analyzing synchronized tapping data (Dalla-Bella & Sowinski, 2015). As a result, the objective assessment of rhythm production can now be conducted with millisecond precision.

However, the psychometric evaluation of the stimulus material is still often under-discussed in rhythm production, particularly when it comes to sequence-based stimuli, such as rhythmic patterns. Only a few studies have systematically examined the psychometric suitability of test materials. Those that have often relied on small sample sizes (Kaplan, 1977). Once published, rhythm patterns are frequently reused in subsequent studies with minimal justification, often referencing only their prior use (Atterbury, 1983). In other cases, rhythmic stimuli are created ad hoc to meet specific “Gestalt” principles relevant to a particular research objective (Drake, 1993; Smith et al., 1994). While it can be reasonably assumed that rhythm reproduction tasks possess high face validity, it remains essential to clarify which target population is being addressed, whether the difficulty level of the stimuli is appropriate, and whether the items constitute a coherent and reliable scale. Recently developed validated test batteries, such as the Battery for the Assessment of Auditory Sensorimotor and Timing Abilities (BAASTA; Dalla-Bella et al., 2016) and the Tapping module of the Profile of Music Perception Skills (Tapping PROMS; Georgi et al., 2023), address these psychometric concerns. However, these instruments were developed and validated for adult populations, and their psychometric properties cannot be presumed to generalize to children across different developmental stages.

## Measuring rhythmic abilities in children

If one aims to assess productive rhythm skills in children, the primary considerations should be an appropriate range of rhythm difficulty and the suitability of the execution modality. While traditional test batteries for measuring rhythm production often rely on finger tapping, such as on touchscreens (DallaBella et al., 2016; Georgi et al., 2023), research has shown that children find clapping or actual drumming easier to perform (Will, 2024). Additionally, the stimulus material should not be overly complex (Reifinger, 2006). In the case of a synchronization task, the stimulus material typically consists of a simple isochronous beat and can only be modified by adjusting the inter-onset intervals (IOI), e.g., making it faster or slower. However, in a pattern-based task, such as rhythm pattern reproduction, task difficulty can be significantly influenced by the complexity of the stimulus pattern. Studies have shown that children memorize and distinguish rhythm patterns more effectively when the underlying beat structure is particularly salient (Nitin et al., 2023). This aspect is also reflected in established theories of rhythm complexity, making them a suitable theoretical foundation for designing stimulus material for children.

## Rhythm complexity

Complexity theories suggest that the perception of rhythm can be understood as an internal timing process, similar to an internal clock, which provides the fundamental structure upon which the rhythm pattern is layered. This internal clock emerges in response to the perceived rhythm and is primarily determined by the impression of accented events. The unit of measurement of the internal clock always corresponds to a multiple of the smallest interval unit (i.e., the shortest distance between two events), yet it may vary between individual listeners. Foot tapping can be interpreted as an overt manifestation of the internal clock. Rhythm patterns differ in extent to which they highlight or induce the feeling of an inner clock. If a rhythm pattern allows for strong clock induction, it is generally perceived as less complex. The strength of clock induction can be quantified and, following Povel and Essens (1985), is referred to as C-score. The induced clock then serves as a reference frame for predicting the elements within the rhythmic pattern, i.e., when the next event occurs. The accuracy of this prediction is a key factor of the overall complexity of a pattern and is taken into account in a measure called coding complexity. It increases when the events in the rhythm pattern are more difficult to predict, e.g., when there is higher uncertainty and ambiguity, due to irregularities. A weighted combination of the C-score (C) and the coding complexity (D) yields the total complexity, which functions as a theoretical indicator of pattern difficulty.

## Rhythm Reproduction Task

In summary, pattern-based rhythm production is a demanding component of rhythmic competence that develops during childhood. Despite its importance, it has received comparatively little attention in the research literature when compared to rhythm perception and beat production. When assessing pattern production in children, the complexity and thus difficulty of the stimulus materials must be carefully considered. Rhythm reproduction is the most commonly standardized measure of pattern-based rhythm production ability. As such, it is also a component of the Music Screening Procedure developed by Jungbluth and Hafen (2005). Recognizing the multimodal nature of musicality and aiming for a more holistic understanding of a child’s ability, this screening incorporated various tasks, including perception, beat-based production, and pattern-based production. For the pattern-based production, the authors of the Music Screening designed ten rhythmic sequences intended to be suitable for children aged 5 to 8 years. Although the test was never officially validated and published, it has been applied on multiple occasions (Degé et al., 2015; Kertész & Honbolygó, 2023; Olbertz, 2006; Sallat, 2012; Steinbrink et al., 2019; Will, 2024). This provides a foundation for further evaluation of the stimulus quality. Will (2024) employed the Rhythm Reproduction Task and further developed three alternatives per item (pattern) for use in experimental designs involving multiple response modalities. Based on this data, we aim to examine the properties of the Rhythm Reproduction Task in greater depth. To this end, we will explore theoretical rhythm complexity, analyze the distribution of individual items across the designated age groups, and assess the scale properties through a factor-analytical examination of the items. Furthermore, to ensure the comparability of the permuted item versions, we aim to establish measurement invariance. This will verify that item sets composed of permuted patterns retain their measuring properties and yield comparable performance results. Finally, given an adequate model fit, we test whether the model holds across all modalities.

## Method

This study presents a secondary analysis of data that partially overlap with those reported in Will (2024). Compliance with ethical guidelines was reviewed by the Ethics Council of the Max Planck Society and confirmed with the vote [2019_25]. Informed consent was obtained from the legal guardians of each participant prior to participation. In addition, the willingness to participate was asked directly from the children before each session. Participation was rewarded with a small gift and a certificate.

## Sample

The study examined *N* = 151 (90 girls, 61 boys) children in daycare centers and elementary schools in the greater Frankfurt and Giessen area (central Germany). The sample covered the ages 5 years (*n* = 29), 6 years (*n* = 29), 7 years (*n* = 47), and 8 years (*n* = 36), for further information see Table 1.

**Table 1:** Demographic sample description

	5-year-old	6-year-old	7-year-old	8-year-old
Age (in months)	*M* = 65.41 (*SD* = 3.51)	*M* = 77.31 (*SD* = 3.01)	*M* = 89.34 (*SD* = 3.80)	*M* = 100.69 (*SD* = 3.35)
Gender	23 f/6 m/0 d	19 f/10 m/0 d	28 f/19 m/0 d	20 f/16 m/0 d
Attend. Music classes (duration in months)	*M* = 1.61 (*SD* = 4.22)	*M* = 2.57 (*SD* = 9.44)	*M* = 2.66 (*SD* = 6.37)	*M* = 2.50 (*SD* = 8.38)
Listening to music (hours per week)	*M* = 3.72 (*SD* = 0.79)	*M* = 3.28 (*SD* = 0.98)	*M* = 3.47 (*SD* = 0.55)	*M* = 3.58 (*SD* = 0.65)
Parental education: University Degree	38 % none 48 % one 14 % both	25 % none 21 % one 54 % both	52 % none 20 % one 28 % both	43 % none 37 % one 20 % both
Family income (per month)	5 % A 54 % B 41 % C	0 % A 46 % B 54 % C	6 % A 76 % B 18 % C	3 % A 59 % B 38 % D
Born in Germany	100 %	97 %	94 %	97 %
Native Language German	72 %	81 %	81 %	87 %

f = female, m = male, d = diverse. Family income per month: A = under 2000€, B = 2000–5000€, C = over 5000€.

## Design

The children completed a rhythm-production task across five conditions. In four conditions, the rhythmic stimulus was presented via a loudspeaker, and the children were instructed to reproduce it by drumming, tapping with a finger, clapping, and vocalizing. In the fifth condition, the stimulus was demonstrated by the experimenter, and the children were required to reproduce it by drumming. To ensure precise and consistent stimulus delivery, the experimenter wore hidden earphones that played the rhythm patterns in real time. The order of conditions was randomized across participants.

## Original Rhythm Reproduction Task

**Fig. 1:**
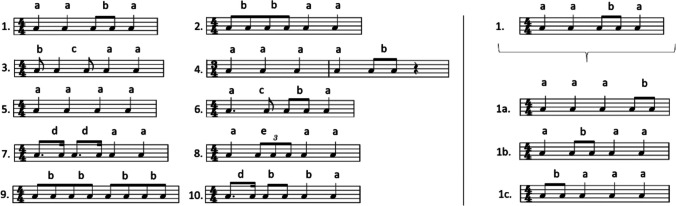
Coding scheme for the original stimulus material with an example of item permutation. *Note*. a = a quarter note; b = two eighth notes; c = syncopated eighth note; d = dotted eighth note followed by a sixteenth note and e = an eighth note triplet

Rhythm reproduction was measured by the Rhythm Reproduction Task of the Music Screening 1 by Jungbluth and Hafen (2005). This task consists of ten rhythmic patterns (items) that are played to the participant, who is then required to reproduce them. All items are set in 4/4 time at 69 BPM and consist of quarter notes, eighth notes, sixteenth notes, dotted notes, syncopations, or a combination of these elements, see Fig. 1. In the original task the stimulus material is presented and also imitated on a keyboard. The original authors intended that a score of 0, 1, or 2 is assigned to each response, depending on whether it was completely incorrect, partially correct, or fully correct. The task begins with an introductory explanation: “You will hear the note A played on a keyboard—play this note using your index finger.” This is followed by an example: “Remember the notes you are about to hear and reproduce them exactly.” The example is structured with a gradual increase in difficulty: “Now, play these notes.” Finally, the participant completes ten test items, introduced with the instruction: “You will now hear ten rhythmic patterns. Try to reproduce the notes as accurately as possible.”

## Adjustments

Please note the following changes compared to the original testing procedure. In the present study, different production modalities were tested and compared. The rhythms were not reproduced using a keyboard but rather through drumming, tapping, clapping, vocalizing, and drumming after experimenter (social). Each participant completed all five conditions, which required the creation of alternative test versions in addition to the original stimuli to avoid learning effects. The creation of the alternatives, as described in the *Alternative Versions* section, required a minor modification of item four. With the aim of making the test more objective and reaching a higher discrimination, the prior scoring has been adjusted in the current analysis. Instead of two possible points per item, a continuous deviation from the presented pattern was calculated. This ensures that the evaluation meets current standards of measurement accuracy.

## Alternative versions

To generate alternative item versions, the stimuli were analyzed with respect to their structural components and subsequently rearranged. Each original rhythm was divided into four elements, which were then permuted in three distinct ways. This resulted in four versions per item (including the original), differing only in the sequence of elements, see Fig. 1. Because items 5 and 9 consisted of identical elements, their permutations were invariant, and these items remained unchanged across all conditions. Item 4 also required special handling, as it already was a modified version of item 1; therefore, an additional note was added, whereby the new stimulus no longer followed a 4/4 but in the current study a 3/4 time. Since the permuted elements align with the segments used for determining complexity, permutation leads only to minor changes in complexity according to Povel and Essens (1985), which is why we consider the permutation approach to be appropriate for these purposes.

## Complexity measure

As defined by Povel and Essens (1985), pattern complexity (*PS*) is composed of clock induction (*C*) and coding complexity (*D*). To estimate clock induction, it is necessary to assume that the pattern is beat-based and therefore underpinned by a latent clock induced in listeners. A higher C-score means less induction, which is why the value could also be referred to as induction difficulty. It is determined by the number of clock ticks that coincide with unaccented events (*u*), as well as those that coincide with silence (*s*). In this computation, clock ticks that fall into silent intervals are weighted by a factor *W*, which should exceed 1, as such mismatches are assumed to hinder clock induction more strongly than the absence of accentuation alone. Based on complexity ratings from an adult sample, Essens (1995) recommends a weighting factor of *W* = 1.17.

C=W·s+u

To calculate the coding complexity D, the rhythmic pattern is divided into individual segments, each corresponding to one clock unit. These segments are then analyzed in terms of predictability of their timing and can be classified into four types: the segment is empty (E); it is evenly subdivided (S); it is unevenly subdivided (U); or it begins with silence (N). The latter two types tend to exhibit lower predictability. Each segment type is assigned a specific weight, reflecting the extent to which that type contributes to the overall complexity. These weights are principally not fixed as they may vary across individuals depending on subjective perceptions of complexity, but for practical purposes, averages can be used. Based on empirical investigations, Essens (1995) proposes the following weights: *d₁* = 0.02 (E), *d₂* = 1.27 (S), *d₃* = 1.30 (U), *d₄* = 0.07 (N). Additionally, the number of transitions between different segment types (denoted as *m*) is counted and weighted as well, with. *d₅* = 0.79. *D* is then calculated as follows, where ${c}_{i}\in \{{d}_{1},\dots ,{d}_{4}\}$ corresponds to the weight of the ${i}^{th}$ segment and n is the number of segments.

$$D=\sum_{i=1}^{n}{c}_{i}+m \cdot {d}_{5}$$

In a final step, the overall complexity value *PS* is computed as a weighted combination of induction strength (*C*) and coding complexity (*D*). Essens (1995) proposes a weight of λ=0.22 to reflect the relative importance of induction strength. For further clarification, an example calculation is provided in the appendix. We use this measure to determine the correlation between complexity and difficulty and thus provide an evaluation of whether it is a suitable basis for item creation.

$$PS= \lambda \cdot C+(1-\lambda )\cdot D$$

## Setup

**Fig. 2:**
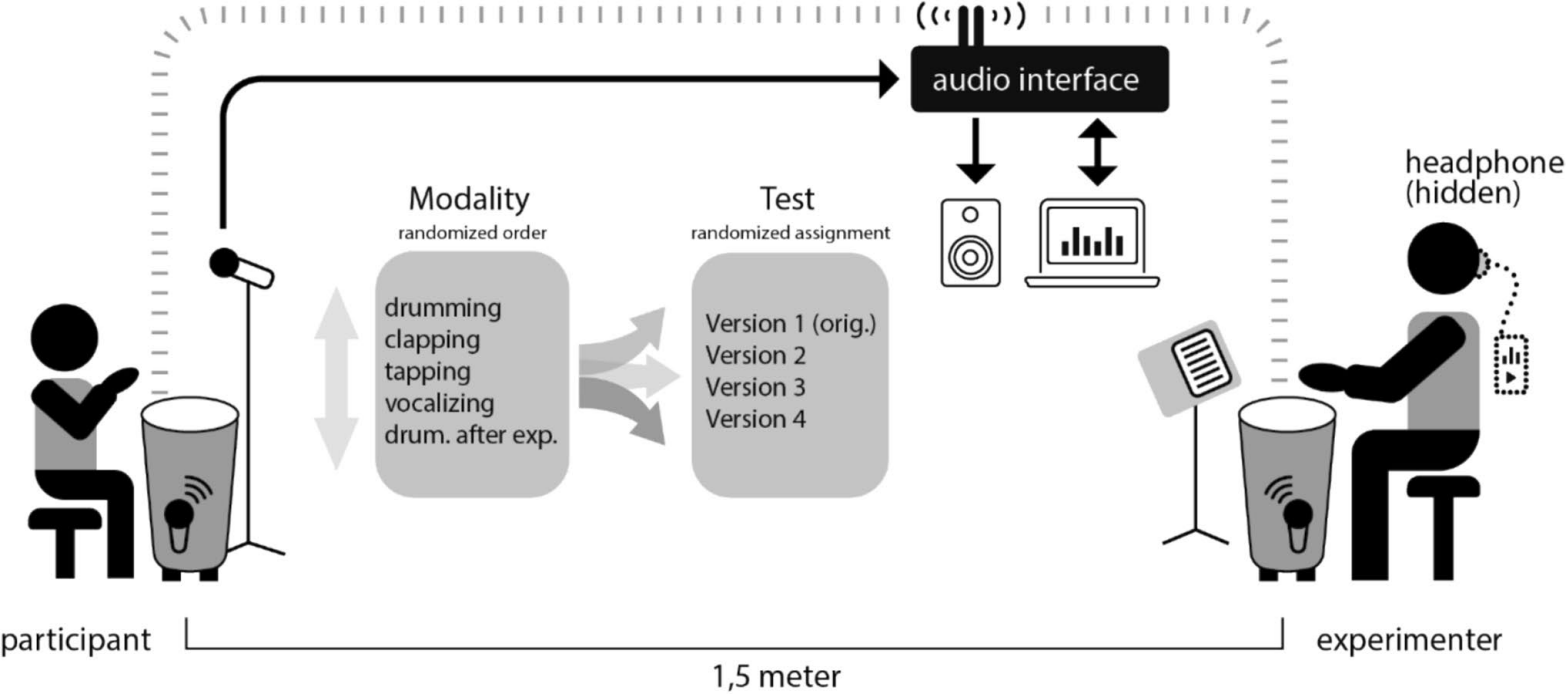
Setup

The tests were conducted in a quiet room within the child’s familiar environment, such as a kindergarten or after-school care facility. In the testing room, two identical drums (height: 56.00 cm, diameter: 25.40 cm) were positioned 1.5 m apart. Each drum was equipped with a wireless microphone that captured the sound directly from the drumhead and transmitted it via a wireless system to an audio interface. For conditions without drumming, a room microphone was used to record the child’s responses, positioned to optimally capture the sound. The stimuli were presented via a loudspeaker at the child’s head level, ensuring high-quality audio playback, see Fig. 2.

## Procedure

The children were tested individually by a trained experimenter. First, the procedure was explained to the child, and their willingness to participate was confirmed. The session began with a short warm-up, during which the children were shown the best way to position their hands, and then, through a playful stop-and-go process, explored different sounds, such as loud, soft, fast, and slow. After that, the reproduction tasks were conducted under five different conditions. In each trial, the rhythms were presented in a predetermined order according to Jungbluth and Hafen (2005). The stimulus was either demonstrated by the experimenter (in the *drumming after experimenter* condition) or played via a concealed loudspeaker. Once the stimulus presentation ended, the experimenter prompted the participants with a hand gesture to reproduce the rhythm. The completion of a single item set (ten items, and one example trial) took an average of *M* = 3.4 min (range 3.0–6.8 min). The full assessment, encompassing all five modalities, lasted an average of *M* = 24.6 min.

## Analysis

**Fig. 3:**
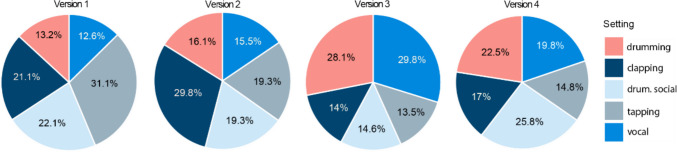
Distribution of modalities in item 1 across permuted versions. *Note:* “drumming” refers to drumming after loudspeaker; “drum. social” refers to drumming after experimenter

Each recording resulted in a single audio file per participant. The recordings were edited using Audacity (Audacity Team, 2021) and subsequently analyzed with the “Vamp Plugin” Note Onset Detector from Queen Mary University (Duxbury et al., 2003) to transcribe the onsets. The corresponding onsets of stimulus and answer (e.g., reproduction on drum) were analyzed for similarity using Dynamic Time Warping (DTW) with the *dtw* package (Giorgino, 2009) in R-Studio. While a normalized distance of 0 would correspond to a perfect reproduction, a greater distance indicates a greater deviation from the target, thus a lower accuracy in the reproduction. In contrast to local distance measures, Dynamic Time Warping (DTW) computes the minimal cumulative distance along an optimal alignment path when comparing two vectors. During this comparison, a value is not required to be matched only with its directly corresponding counterpart; instead, it may be aligned with a preceding or subsequent reference value, depending on which yields the best fit. If a child plays too few or too many beats, reference beats must be aligned multiple times, increasing the amount of warping required and typically leading to a higher overall distance. The 151 participants completed five production conditions (drumming with a metronome, drumming with an experimenter, clapping, tapping, vocalizing), each involving ten items. This resulted in a total of *N* = 7550 processed items. Missing values were treated as missing completely at random and handled using full-information maximum likelihood. Since there were five experimental conditions but only four permuted item sets, one of the item sets was randomly repeated in the final condition. Consequently, for each participant, certain items were observed twice. For the purpose of item analysis, these duplicate observations were averaged. It should be emphasized that the data analyzed for each item represent aggregated outputs from various production modalities. Due to repeated presentations in the final condition, the relative contributions of these modalities within individual items differ slightly. This should be taken into account when conducting comparative analyses of corresponding items across different versions. For example, when comparing the difficulty of item 1 across different test versions, the unequal distribution of settings introduces an error, as the settings themselves also vary in difficulty (see Fig. 3).

## Results

Overall reproduction performance was determined by the average scores of all test items across all production conditions. On average, overall performance was *M* = 0.25 (*SD* = 0.19), with a minimum of 0.005 (best score) and a maximum of 2.112 (worst score). As the values are normalized through DTW distance computation, they lack direct absolute interpretability. The distribution of normalized distances across all items represents a deviation with a natural lower bound at 0 and an unbounded upper limit, due to this, the distribution is skewed to the right and a slight floor effect is present. Therefore, the distances are logarithmized for further analyses. Logarithmization shifts good values (originally close to 0) into the negative value range, so more negative values are henceforth considered better compared to values close to 0.

**Fig. 4:**
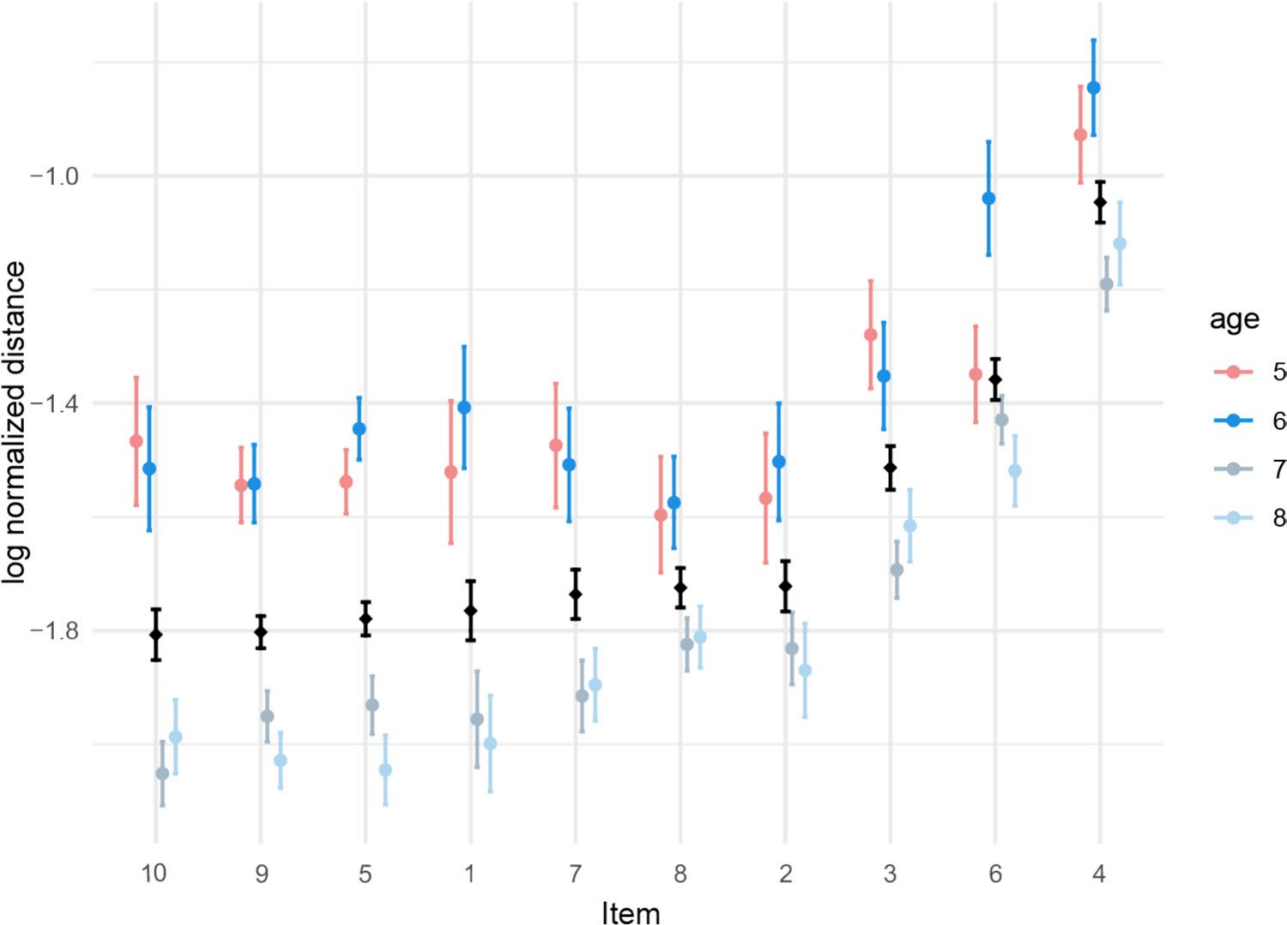
Log-normalized distance per item, illustrating the recommended item order for ascending difficulty. *Note*: *Black bars* indicate the mean distance across age groups. *Error bars* represent ±1 standard error of the mean

When analyzing the log-transformed values of the original items across the full sample of children, item 10 displays a mean of *M* = – 1.81 with a standard deviation of *SD* = 0.59. This represents the highest reproduction accuracy and indicates that, within this sample, item 10 was the least challenging. In contrast, item 4 yields a mean of *M* = – 1.04 (*SD* = 0.47), marking the lowest reproduction accuracy and thus appears as the most difficult item (see Table 2). Although the majority of the observed item means are relatively similar, items 3, 6, and 4 show a distinct gradient of increasing difficulty, an observation that applies to all age groups. To enhance engagement with the task, a gradual increase in item difficulty is desirable. Therefore, based on the present data, a reordering of the items is recommended, as illustrated in Fig. 4.

**Table 2:** Item reliability statistics

Item	Mean	SD	*PS*	Item-total correlation^1^	If item dropped	
					Cronbach‘s α	McDonald’s ω^2^
1 - aaba	– 1.79	0.66	6.189	0.51	0.81	0.59
2 - bbaa	– 1.75	0.52	5.532	0.43	0.81	0.73
3 - bcaa	– 1.51	0.50	6.210	0.51	0.81	0.62
4 - aaaab	– 1.04	0.47	7.973	0.46	0.81	0.65
5 - aaaa	– 1.76	0.54	4.991	0.65	0.79	0.64
6 - acba	– 1.34	0.45	6.096	0.41	0.81	0.58
7 - ddaa	– 1.74	0.52	5.579	0.54	0.80	0.63
8 - aeaa	– 1.74	0.41	6.655	0.36	0.82	0.70
9 - bbbb	– 1.80	0.48	4.840	0.73	0.78	0.57
10 -dbba	– 1.81	0.59	6.134	0.49	0.81	0.73

^1^Corrected, ^2^ω Hierarchical, *PS* = Complexity score

**Fig. 5:**
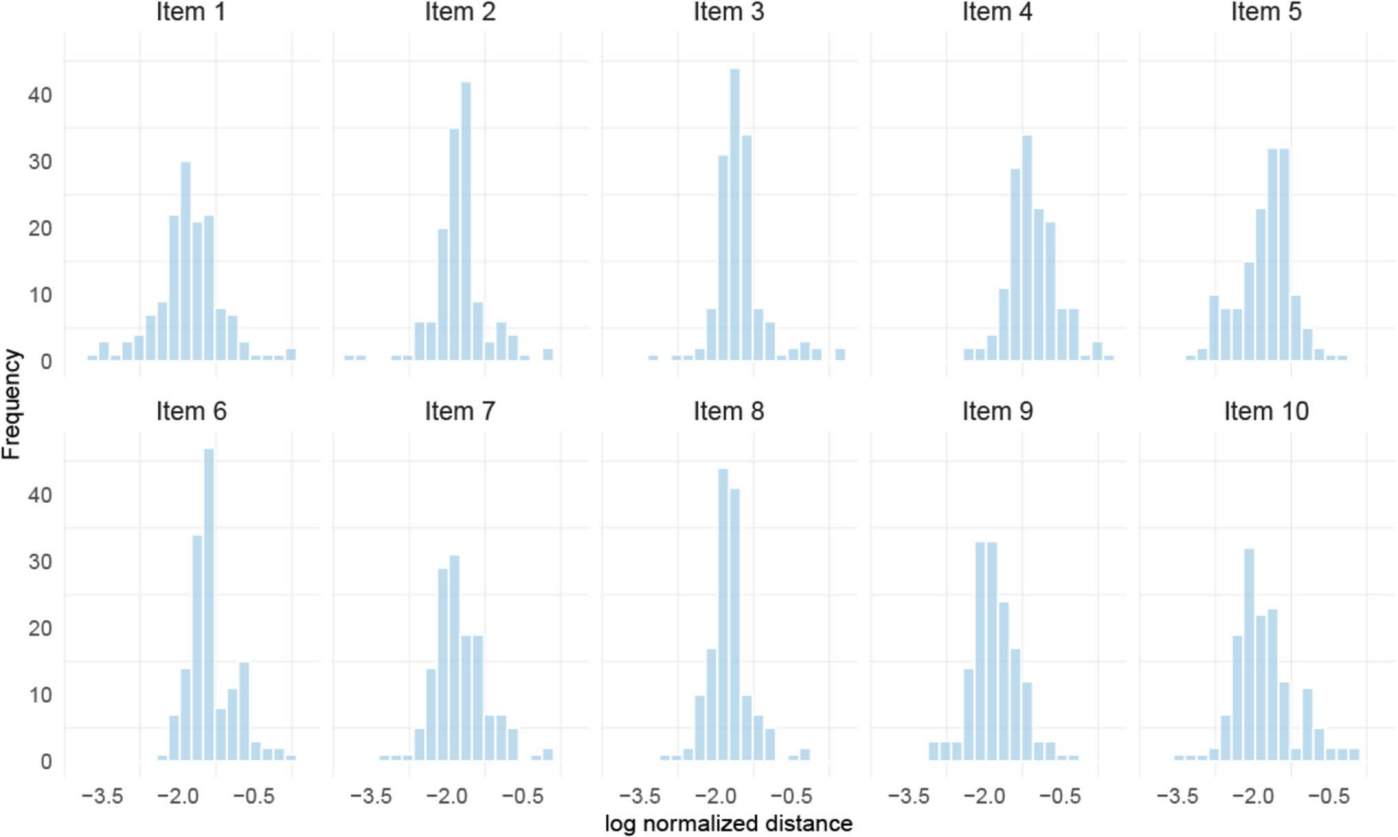
Histograms of the reproduction accuracy for all items in the original version. *Note:* Frequency refers to number of children (${N}_{total}$= 151)

Item 8 shows the lowest standard deviation (*SD* = 0.41), while the results on item 1 are the most scattered (*SD* = 0.61). All items show a visible variance, which indicates a differentiated representation of the measured ability (see Fig. 5). The internal consistency of the scale, estimated by Cronbach’s alpha, is 0.82 (95% CI [0.78, 0.86]). Item 8 exhibits the lowest corrected item-total correlation (${r}_{it}$ = 0.36), whereas item 9 shows the highest (${r}_{it}$ = 0.73). Thus, the majority of items fall within the recommended range of 0.40 to 0.70 and can be considered acceptable to good according to Moosbrugger and Kelava (2012). Individuals with high overall rhythm reproduction ability also tend to score higher across individual items. Notably, the exclusion of any item does not improve internal consistency.

The theoretical item complexity (*PS*) was calculated according to Povel and Essens (1985), using the recommended weights provided by Essens (1995) namely λ = 0.23; *W* = 1.17; ${d}_{1}$= 0.02; ${d}_{2}$ = 1.27; ${d}_{3}$ = 1.29; ${d}_{4}$ = 0.07; ${d}_{5}$ = 0.79. Based on this assessment, the item with the lowest complexity is item 9, with a score of 4.84, while the highest theoretical complexity is found in item 4, with a score of 7.97. These two extreme positions are close to the perceived difficulty of the items, but do not correspond to the order proposed by Jungbluth and Hafen (2005). The ranking of items between these extremes differs from both the observed difficulty and the original sequence. The Spearman rank order correlation between the observed distances and the theoretical item complexity (Povel & Essen 1985) for all items in the extended item pool is 0.29 (*p* = 0.097). The correlation is therefore moderately positive but not significant in this analysis.

## Factor analysis

To further assess the scale properties, a unidimensional linear model was specified with the latent factor *rhythmic reproduction ability *(see Table 3). To ensure identifiability, the loading of the easiest item was fixed to 1. Under the assumption of residual independence, the model demonstrated an acceptable fit with *χ*^*2*^ (35) = 66.85 and the fit indices RMSEA = 0.078, SRMR = 0.059, and CFI = 0.92. However, modification indices suggested allowing error correlations between items 1 and 2 as well as between items 1 and 5. Incorporating these adjustments resulted in Δ *χ*^*2*^ (2) = 18.08, confirming a significantly improved model fit with *p* <.001. This error correlation may be attributed to an unequal distribution of the setting condition and was therefore deemed justifiable. The revised fit indices were RMSEA = 0.056, SRMR = 0.059, and CFI = 0.96. All factor loadings were statistically significant at the 0.01 level, with standardized loadings ranging from 0.44 for item 6 to 0.84 for item 9. For additional values, please refer to Table 4. Notably, all factor loadings exceeded the critical threshold of 0.3, indicating moderate to high loadings (Moosbrugger & Kelava., 2012). Omega total is high at 0.80, and the average variance extracted (AVE) for the latent factor is moderate at 0.33.

**Table 3:** CFA model fit for the original test items

Model	*n*	Chi-square (*df*)	*p* value	RMSEA	SRMR	CFI	TLI
1 factor	150	66.845 (35)	.001	0.078	0.059	0.92	0.89
1 factor*	150	48.763 (33)	.038	0.056	0.052	0.96	0.94

* Residual correlation between aaba-bbaa and between aaba-aaaa

**Table 4:** CFA – 1 factor solution

Item	Estimate	*SE*	*Z*	*p* value	Stand. estimate
1 - aaba	0.980	0.214	4.576	<.001	0.483
2 - bbaa	0.794	0.179	4.443	<.001	0.487
3 - bcaa	0.867	0.165	5.259	<.001	0.564
4 – aaaab	0.751	0.154	4.868	<.001	0.519
5 - aaaa	1.133	0.192	5.914	<.001	0.688
6 - acba	0.608	0.141	4.300	<.001	0.437
7 - ddaa	1.032	0.183	5.625	<.001	0.637
8 - aeaa	0.592	0.135	4.377	<.001	0.467
9 - bbbb	1.227	0.190	6.447	<.001	0.840
10 -dbba	1.000*	-	-	-	0.550
1.aaba ~~ 2.bbaa	0.070	0.021	3.260	.001	0.312
1.aaba ~~ 5.aaaa	0.070	0.024	2.944	.003	0.262

*fixed parameter (easiest item)

**Fig. 6:**
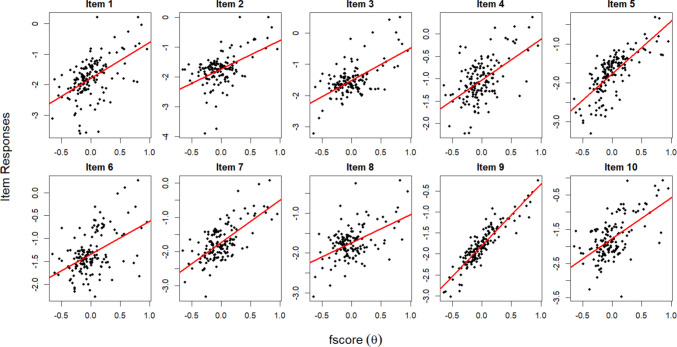
Log-normalized distance per item plotted against model-predicted ability parameters

To gain a more precise understanding of the individual items, the model-implied factor score was estimated for each child. This score was plotted against performance on the individual test items (see Fig. 6). A slight clustering of the predicted latent ability values is observable. All item performances exhibit a positive relationship with the factor score. As evident in Fig. 6, item 9 demonstrates the highest discriminatory power, whereas item 8 stands out with a slightly flattened regression slope.

## Investigation of the comparability of alternating item versions

**Fig. 7:**
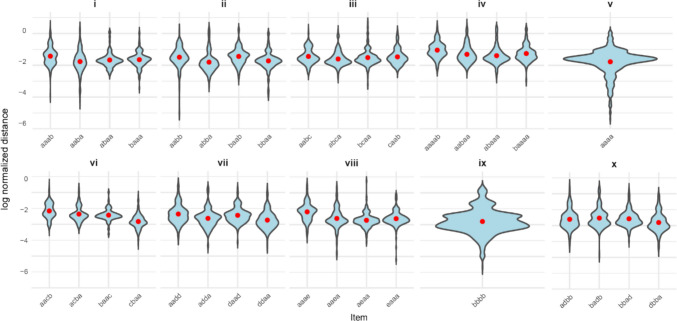
Violin plot of responses across permuted item versions. *Note:* Mean distance across age groups is shown for comparison of corresponding item versions (Roman numerals indicate the respective position in the original test, e.g., item 1 of test version 1, item 1 of test version 2, etc.)

From the original stimulus material, three comparable versions of each rhythm were created by rearranging individual pattern elements. These alternative versions were constructed to enable direct comparisons across different modes of execution and were designed to be as equivalent as possible in terms of their capacity to assess rhythmic reproduction skills. Descriptive comparisons of mean performance values across the different item versions revealed some notable differences; in some cases, the permutation resulted in slightly easier tasks, while in others, it led to increased difficulty (see Fig. 7). However, the theoretical complexity score (*PS*) remained largely consistent across all versions, as the rearranged elements corresponded to the same segments used for determining rhythmic complexity. Thus, any variation in complexity was limited primarily to the number of transitions between elements rather than to the structural features of the patterns themselves.

To allow the original item set to be adapted for varying production conditions, three additional sets were assembled from the expanded item pool for use in the present study. The log-transformed total reproduction performance across the different item sets consistently demonstrated strong and statistically significant Pearson product–moment correlations (Pearson, 1904). Specifically, the original test correlated at *r* =.86 (95% CI [.80,.90]) with Set 2, *r* =.86 (95% CI [.80,.90]) with Set 3, and slightly lower at *r* =.84 (95% CI [.77,.88]) with Set 4. The correlation between Set 2 and Set 3 was *r* =.88 (95% CI [.82,.91]), between Set 2 and Set 4 was *r* =.89 (95% CI [.85,.92]), and between Set 3 and Set 4 was *r* =.87 (95% CI [.82,.91]). These findings suggest that strong performance on one version of the test is highly likely to be associated with strong performance on several other permuted test versions.

To provide a comprehensive picture and to assess structural comparability, all 34 items were analyzed jointly in the following section by integrating them into a single measurement model. A parallel analysis was conducted to determine the appropriate number of factors, which—consistent with expectations—indicated a single underlying factor. The internal consistency of the full scale was high, with Cronbach’s alpha =.94 (95% *CI* [.93,.95]) and omega total =.94. A unidimensional model, in which all items loaded onto a single latent factor and residual correlations were allowed between corresponding item versions, demonstrated an acceptable yet improvable fit to the data: *χ*^*2*^ (486) = 902.34, RMSEA =.076, SRMR =.069, and CFI =.80. Allowing for additional correlated error terms between selected items (see Table 5) led to a slight but statistically significant improvement in model fit: *χ*^*2*^ (479) = 798.77, RMSEA =.067, SRMR =.065, and CFI =.85. Standardized factor loadings ranged from.400 for pattern *cbaa* (Version 2, Item 6) to.774 for pattern *bbbb* (Item 9). The average variance extracted (AVE) by the latent factor was.35. The fit indices indicated a mixed model fit. In particular, the CFI did not reach the desirable threshold of .90. As noted earlier, the unequal distribution of production formats across the item comparisons may provide a plausible explanation for residual correlations. Given the acceptable to good values of the RMSEA and SRMR, we conclude that the assumption of configural invariance across permuted item versions is met. In a next step, factor loadings of all corresponding item permutations were constrained to be equal. This resulted in a marginally acceptable model fit: *χ*^*2*^ (503) = 836.16, RMSEA =.066, SRMR =.092, and CFI =.84. Modification indices suggested freeing the loading of *aaea*, a variant of item 8. This adjustment led to a statistically significant improvement in model fit, Δ *χ*^*2*^ (1) = 7.52, *p* =.006, resulting in a revised model with *χ*^*2*^ (502) = 828.64, RMSEA =.066, SRMR =.086, and CFI =.84. The fit of the model with equal loadings no longer differed significantly from the general model, Δ *χ*^*2*^ (23) = 29.87, *p* =.153. These results support the assumption of partial scalar invariance. An additional constraint equating the corresponding item intercepts resulted in a significant deterioration of model fit, Δ *χ*^*2*^ (1) = 7.27, *p* =.007. This difference could only be accommodated by freely estimating the majority of the intercepts, thereby precluding the assumption of scalar invariance and providing little support even for partial scalar invariance.

**Table 5:** Model fit statistics for battery and metric measurement invariance model

Model	*n*	Chi-square (*df*)	*p* value	RMSEA	SRMR	CFI	TLI
1 factor	150	902.34 (486)	<.001	0.076	0.069	0.80	0.77
1 factor*	150	798.77 (479)	<.001	0.067	0.065	0.85	0.82
Metric - partial^1^	150	828.64 (502)	<.001	0.066	0.086	0.84	0.83

* Residual correlation between items badb-aacb; bcaa-aadd; abaaa-abba and aaaa-caab; ^1^ free loading aaea

The metric invariance across the different item permutations indicates that the respective item variations are equally influenced by the latent construct of rhythmic reproduction ability. This suggests that the items are comparable in their quality as indicators of this construct. This provides a basis for combining the various item versions used to test the production modalities and, in turn, for comparing these modalities with respect to metric invariance. Such a comparison allows us to determine whether the factor structure and the suitability of the items are comparable, depending on whether the test is administered through drumming, tapping, clapping or vocalizing. First, each modality was examined separately using a single-factor model to assess the basic model fit, which was good across most production modalities only reduced for finger tapping (see Table 6A). Invariance testing was then conducted by combining two modalities at a time within a model comprising two correlated factors. The residuals of corresponding items were allowed to correlate, and in the next step, the factor loadings of the corresponding items were constrained to be equal. It should be noted that a simultaneous analysis of all modalities within a single model was not feasible due to the limited sample size.

**Table 6:** A. Single-factor model fit results by modality

	*n*	Chi-square (*df*)	*p* value	RMSEA	SRMR	CFI	TLI
drumming (dr)	150	71.802 (35)	<.001	0.086	0.057	0.91	0.88
clapping (cl)	150	46.951 (35)	0.085	0.048	0.042	0.98	0.97
tapping (ta)	150	94.967 (35)	<.001	0.109	0.061	0.88	0.84
vocalizing (vo)	150	37.082 (35)	0.373	0.027	0.027	0.99	0.99
drum. social (so)	150	47.052 (35)	0.084	0.048	0.050	0.95	0.94
B. Fit indices: Metric measurement invariance between modalities							
dr vs. cl	150	264.56 (168)	<.001	0.062	0.071	0.91	0.90
dr vs. ta	147	329.07 (168)	<.001	0.081	0.080	0.84	0.82
dr vs. so	148	249.85 (168)	<.001	0.057	0.075	0.89	0.88
dr vs. vo	149	216.80 (168)	0.007	0.044	0.065	0.96	0.96
cl vs. ta - partial^1^	150	262.16 (167)	<.001	0.062	0.075	0.92	0.91
cl vs. so	150	210.15 (168)	0.015	0.041	0.076	0.95	0.95
cl vs. vo - partial^2^	147	222.89 (167)	0.003	0.048	0.058	0.96	0.96
ta vs. so	147	238.30 (168)	<.001	0.053	0.062	0.92	0.91
ta vs. vo - partial^3^	149	245.09 (165)	<.001	0.057	0.072	0.94	0.93
so vs. vo - partial^2^	149	203.40 (167)	0.029	0.038	0.070	0.97	0.97

^1^ free loading 8, ^2^ free loading 6, ^3^ free loading 2, 5 and 6; “drumming” refers to drumming after the loudspeaker; “drum. social” (so) refers to drumming after experimenter

For the majority of modality comparisons, metric invariance could be established without difficulty, as constraining the factor loadings did not result in a notable deterioration of model fit (see Table 6B). A few exceptions emerged: In the comparison between *clapping* and *tapping*, constraining the loadings led to a poorer model fit, Δ *χ*^*2*^ (9) = 24.24, *p* =.003, which was resolved by freely estimating loading 8. Similarly, the fit of the *clapping* and *vocalizing* model deteriorated under loading constraints, Δ *χ*^*2*^ (9) = 18.63, *p* =.029; freeing loading 6 addressed this difference. The *tapping* and *vocalizing* model also deviated from the assumption of equal loadings, Δ *χ*^*2*^ (9) = 23.46, *p* =.005. In this case, the equality constraints for items 2, 5, and 6 had to be released in order to match the fit of the model with freely estimated loadings. Lastly, the comparison between *drumming after experimenter* and *vocalizing* showed a significantly worse model fit when loadings were constrained, Δ *χ*^*2*^ (9) = 19.61, *p* =.021. Here, too, freeing loading 6 was sufficient to align the model fit with that of the unconstrained model. For all resulting model fits, see Table 6.

## Test scores

**Fig. 8:**
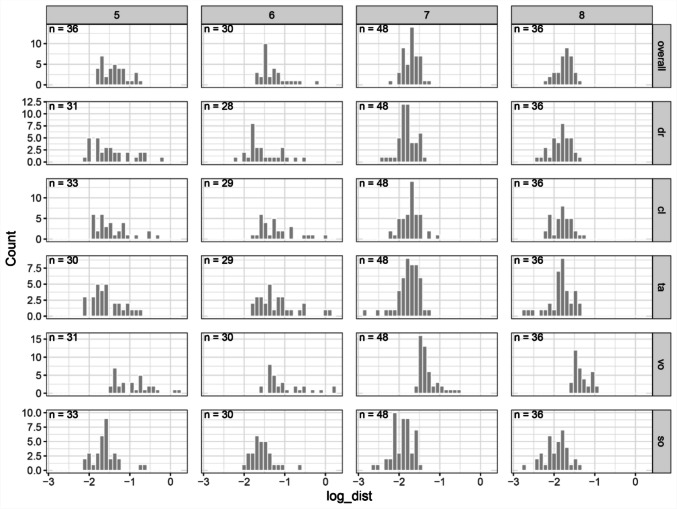
Distribution of test scores for age groups and modalities. *Note.* Overall = across modalities, dr = drumming, cl = clapping, ta = tapping, vo = vocalizing, so = drumming after experimenter

Finally, we move beyond the item level to examine the distribution of test scores within each age group and testing condition. Overall, test scores approximate a normal distribution, with increasing age and sample size, see Fig. 8. The broad score range suggests sufficient individual differences to detect meaningful variation. Visual inspection reveals broadly comparable distributions, with slight deviations in the drumming-after-experimenter condition (perceived as easier) and the vocalizing condition (perceived as harder).

The observed mean distance revealed moderate to high significant positive correlations across modalities (see Table 7). Performance on the same test varied depending on the mode of execution, despite generally strong inter-modal consistency. These findings replicate earlier evidence from Rainbow (1981) and underscore the importance of a differentiated assessment approach, supporting the use of parallel test versions to ensure valid and reliable measurement across modalities.

**Table 7:** Correlation of mean distance between modalities

	Total	ac	cl	so	ta	vo
total	1					
drumming (dr)	.87*	1				
clapping (cl)	.91*	.76*	1			
drum. social (so)	.82*	.62*	.66*	1		
tapping (ta)	.88*	.71*	.78*	.69*	1	
vocalizing (vo)	.77*	.60*	.63*	.51*	.58*	1

* *p* <.001; “drum. social” (so) refers to drumming after experimenter

## Discussion

The present study aimed to evaluate the psychometric quality of the Rhythm Reproduction Task in children aged 5 to 8 years. Subsequently, we examined whether this quality also extends to permuted versions of the original items. In this context, the theoretical rhythm pattern complexity proposed by Povel and Essens (1985) was incorporated and analyzed in relation to the empirically observed item difficulties. Finally, the factor structure of the task was examined across different production modalities.

The stimulus material used in the Rhythm Reproduction Task of the Music Screening Procedure by Jungbluth and Hafen (2005) demonstrates strong overall psychometric properties. All items show substantial response variability and appropriate levels of difficulty. Notably, the items vary in difficulty, which is desirable for capturing a broad range of rhythmic ability.

However, the intended progression of difficulty in the original item sequence did not correspond to the empirically observed item difficulties, nor did it align with expectations based on theoretical rhythm complexity. A striking example is item 9, which, although designed to be more difficult due to its composition of eight eighth notes, was reproduced more accurately than item 5, which consists of four quarter notes. This counterintuitive result may be explained by the concept of spontaneous motor tempo (SMT)—an individual’s natural and preferred rate of rhythmic movement. Motor performance tends to improve when task tempo aligns with SMT (Monier & Droit-Volet, 2018). For adults, SMT averages around 100 beats per minute (bpm) (Desbernats et al., 2023), considerably faster than the 69 bpm used in the test stimuli. This effect is likely even more pronounced in children, whose SMT is typically higher than that of adults (Provasi & Bobin-Bègue, 2003). Accordingly, a sequence of eighth notes may be easier for children to reproduce than a slower sequence of quarter notes, as it more closely aligns with their natural motor rhythm.

Additionally, Item 10—the final item in the sequence—was among the easiest, suggesting a potential sequence effect arising from the fixed item order. Children’s performance may have improved toward the end of the task due to increasing familiarity, practice, or motor warm-up. However, we consider this influence to be minimal, as participants completed the approximately 3-min task with the same item order five times in succession. Sequence effects, if present, would therefore likely affect the entire final block rather than item 10 in isolation. Moreover, since the order of test versions and modalities was randomized across trials, any such effects would be evenly distributed across items, minimizing their impact on the relative difficulty ranking of individual items.

The most challenging item was item 4, which had been modified by the authors to allow for restructuring in alternative versions. This adaptation involved adding an extra quarter note, thereby lengthening the stimulus and shifting the time signature from a regular 4/4 meter to a 3/4 meter. Possible explanations for the increased difficulty include the greater stimulus length, the less familiar meter, or the cognitive load associated with an abrupt change in metrical structure. Nevertheless, this modification strengthens the overall scale by extending its difficulty range, facilitating finer differentiation among children with higher rhythmic ability.

In sum, a more finely graded progression of item difficulty would benefit the original item set. A reordering of item presentation is therefore recommended.

The test scale demonstrated high internal consistency, with most items contributing to the measurement accuracy of a single latent construct. Only item 8 showed slightly lower scale fit compared to the other items across the tested metrics. This may be due to its distinctive nature, as it is the only item that includes an eighth-note triplet, which sets it apart from the other rhythms. However, it also broadens the range of rhythmic reproduction demands and can therefore be considered a meaningful enhancement. The test is well represented by a single-factor model with good model fit. The average explained variance is moderate. This is most likely due to substantial error variance, possibly resulting from the previously mentioned uneven distribution of production modalities, or simply reflecting a common and expected limitation in studies involving young children, which are susceptible to disturbances.

The preceding analyses aimed to evaluate the comparability of the original test items with each of the newly developed item versions, as well as the comparability among these alternative versions. The observed item means showed descriptive differences. However, the items could be easily combined within a single-factor model. This suggests that the newly generated rhythmic stimuli integrate well into the scale established by the original rhythms, reinforcing the notion that all item performances reflect a shared latent construct, referred to here as *rhythmic reproduction ability*. The model required a slight relaxation of the assumption of independent residuals for specific item pairs. These modifications were deemed appropriate, as it is reasonable to assume that certain items exhibited similarities beyond the latent ability—particularly due to shared structural features resulting from the distribution of rhythmic parameters, which created comparable task conditions. A key finding is that partial metric invariance was achieved, with only one of the 34 factor loadings requiring release from the equality constraint. This indicates that the corresponding item versions were influenced by the latent ability factor to a comparable degree. However, scalar invariance proved more difficult to establish, as equality constraints on item intercepts could be upheld for only a limited subset of items. This finding is consistent with the observed differences in item means across versions. The permutation of individual rhythmic elements does not appear to automatically produce rhythm patterns of equivalent difficulty.

This observation can be explained only to a limited extent by the theoretical complexity values. These values are derived from analyses of individual rhythmic segments and change only slightly with permutations, provided that the permuted elements correspond to rhythmic segments, as assumed in the present study. This assumption relies on the premise that the clock induction, as quantified by the C-score, remains stable across permutations. However, this assumption is subject to uncertainty, as certain permutations may yield rhythms with more ambiguous clock induction. For example, depending on the specific permutation, the first clock tick may align with either the first or the second event. Based on theoretical considerations alone, it is not possible to determine with certainty which internal temporal structure—or “internal clock”—was actually perceived by a given participant. This uncertainty is further amplified by the brevity of the presented rhythms, which were not repeated during the task. Additional limitations arise regarding the segment weights used in the complexity calculations, as they were derived from an adult sample. It remains unclear whether children may assign different salience to rhythmic features, potentially perceiving certain elements as more disruptive, or whether individual differences in salience attribution exist more broadly. As such, the complexity index should be regarded as a rough approximation of rhythmic complexity. Nevertheless, it provides a useful estimate that aligns reasonably well with the observed performance data.

Despite the descriptive differences observed at the item level, a consistently high degree of comparability was found in overall performance across the different item sets. Therefore, it can be concluded that an individual’s total score is highly comparable across test versions.

Since equal factor loadings were observed for nearly all items, the item versions were combined in a final step to enable comparison of the factor structure across the tested modalities—drumming, clapping, tapping, and vocalizing. Most modalities demonstrated good model fit and high reliability, with only a slightly poorer result for finger tapping. Model fit for separated modalities even exceeded that of the previously tested models in which modalities were mixed within a single item. These findings strengthen the assumption that the uneven distribution of modalities contributed to increased error variance, potentially leading to an underestimation of model fit in the previous analyses. The factor loadings of individual items were largely consistent across modalities, indicating that each item’s reflection of the latent ability is independent of the specific modality through which it is expressed. Consequently, the measurement quality of the test appears to be comparable across different modes of rhythmic production.

## Limitations and future outlook

While this study offers valuable insights into test quality, several limitations should be acknowledged. Although it has been shown that short-term memory accounts for only about 10% of the variance in pattern reproduction among adults (Grahn & Schuit, 2012), the relationship to working memory should be examined, particularly for the target group of children, in order to ensure discriminant validity. This is an endeavor that will be carried out in a comprehensive manner in subsequent projects. Furthermore, the item identified as the most difficult (item 4) likely owes this status to its extended structure, which was required to enable permutation. In contrast, items 5 and 9 could not be permuted due to their structural properties and were therefore presented in identical form across all conditions. Their repeated appearance may have introduced learning effects, which would justify a cautious interpretation of their high discriminatory power. Additionally, the distribution of item versions across the different response modalities was not fully balanced. Given the observed differences in difficulty between modalities, this may have affected the comparability of item versions, potentially leading to an underestimation of invariance. Based on our findings, a recommended next step is to collect additional data across all item versions in a single modality. This would allow for the selection of an optimized item set from the full pool, which could then be published, with corresponding standard values, in both full and short test formats or used to create a computerized adaptive version to achieve optimal reliability.

## User recommendation

The Rhythm Reproduction Task is a reliable tool for assessing pattern reproduction accuracy in children aged 5–8. The original ten items are sufficient for accuracy-focused assessments. We recommend ordering items by increasing difficulty and using clapping or drumming for optimal model fit. For cross-modal comparisons or repeated administration, the permuted item-sets serve as valid alternative test versions.

## Conclusion

The stimulus material of the Rhythm Reproduction Task from the Music Screening Procedure developed by Jungbluth and Hafen (2005) demonstrated empirically adequate psychometric properties. The test appears well-suited for assessing the targeted ability in children aged 5 to 8 years across various production modalities. Overall test scores derived from the use of permuted item alternatives showed high comparability with those of the original version, indicating their suitability for use in comparative assessments.

## Data Availability

All data, experimental code, and materials are available under https://osf.io/rnkbz/

## Appendix

To illustrate the application of the PS-Measure, consider a rhythmic pattern in 4/4 meter, expressed in binary notation as 10110010. In this notation, ‘1’ denotes a sound event, and ‘0’ denotes a silence or the absence of an event.

The first step is to identify the best-fitting clock—the periodic framework that aligns most consistently with the onsets in the rhythm. Suppose the optimal clock has a unit length of 2 and begins at position 1, yielding clock ticks at positions 1, 3, 5, and 7. Analyzing the alignment between this clock and the rhythm, we observe the following: One clock tick (at position 5) coincides with a silence. Two events (at positions 4 and 6) fall outside the clock ticks. Given these values, and assuming a weighting factor of *W*=1.17, the induction strength of the clock is computed as follows:

$$C=W\bullet s+u=1.17 \cdot 1+2=3.17$$

where *s* is the number of silent clock ticks and *u* the number of unaccented events. Next, we evaluate the coding complexity by segmenting the rhythm according to the clock. The pattern is divided into four 2-bit segments: 10, 11, 00, and 10. Each segment is then categorized based on the structural types defined in the PS-Measure framework. The segments 10 and 11 are both classified as equally subdivided (*S₂*), the segment 00 is classified as empty (*E*). Thus, the segment classification is as follows: S, S, E, S. Each segment is assigned a weight based on its structural classification: ${d}_{1}=0.02$ for *E*, ${d}_{2}=1.27$ for *S*. Thus, the total segment weight is:

SegmentWeight=0.02+1.27+0.02+1.27=2.58

To account for segment transitions, we count the number of structural changes between consecutive segments. In this example, the third segment (*E*) differs from both the second and the fourth (*S*), yielding: *m*=2 (segment transitions). Assuming a transition penalty of ${d}_{5}=0.79$, the coding complexity *D* is calculated as:

$$D=Segment Weight+m \cdot {d}_{5}=2.58+2\cdot 0.79= 4.16$$

Finally, the overall PS-Measure is computed by combining the clock induction strength $\left(C=3.17\right)$ and the coding complexity $\left(D=4.16\right)$, weighted by a factor λ=0.22.

$$PS= \lambda \cdot C+\left(1-\lambda \right)\cdot D=0.22\cdot 3.17+0.78\cdot 4.16=3.92$$
